# Characterization and phylogenetic analysis of the complete chloroplast genome of *Cordia subcordata* Lamarck 1899, a protected plant in China

**DOI:** 10.1080/23802359.2025.2519220

**Published:** 2025-06-20

**Authors:** Mingzhong Liu, Hui Zhang, Wen Tang, Zhenyang Gao, Muqiu Zhao, Yunfeng Shi

**Affiliations:** ^a^ Yazhou Bay Innovation Institute of Hainan Tropical Ocean University, Sanya, China; ^b^ Key Laboratory for Coastal Marine Eco-Environment Process and Carbon Sink of Hainan province, Hainan Tropical Ocean University, Sanya, China

**Keywords:** *Cordia subcordata*, chloroplast genome, phylogenetic analysis, coastal plant, tropic

## Abstract

This study provides the first comprehensive analysis of the chloroplast genome of *Cordia subcordata*, a protected species in China, native to Malesia and widely distributed along the Pacific and Indian Oceans. The genome is 154,811 bp long, circular, with a 38.01% GC content, encoding 133 genes: 88 protein-coding, 37 tRNA, and 8 rRNA genes. Phylogenetic analysis reveals it clusters with other Cordia species and is distinct from genera like *Ehretia* and *Heliotropium*, highlighting significant diversification within the Boraginaceae family.

## Introduction

The genus *Cordia* (Boraginaceae) comprises flowering tree and shrub species primarily distributed across tropical and subtropical regions, including East Africa, Mexico, the West Indies, Central and South America, Pakistan, West Africa, Nigeria, Ghana, Sri Lanka, and India (Verdcourt 1991). Early botanical studies on *Cordia* focused on its reproductive characteristics, highlighting that while these plants are fertile, they are incapable of self-fertilization—an important criterion for taxonomic classification within the genus (Bawa 1974; Opler et al. 1975). Many *Cordia* species are cultivated for ornamental, timber, and medicinal purposes, playing a significant role in traditional communities. Due to their ethnobotanical and ethnopharmacological importance, particularly in the Americas, Asia, and Africa, *Cordia* species have been widely studied. In recent decades, scientific interest in the genus has increased, particularly in its phytochemical, biological, and pharmacological properties (Matias et al. 2015).

The genus *Cordia* (Boraginaceae) includes 271 species, among which *Cordia subcordata* Lamarck 1899 is a notable member (https://www.worldfloraonline.org/). *C. subcordata* is an evergreen shrub or tree that can reach heights of up to 15 meters. Its leaves are ovate to broadly ovate (6–20 × 5–16 cm) and may be either glabrous or covered with minute appressed hairs. The species produces hermaphroditic flowers arranged in cymose inflorescences, and its fruits are smooth and subglobose (https://powo.science.kew.org/). It typically thrives in oceanic islands and dry biomes (Wang et al. 2020).

Globally, *C. subcordata* is widely distributed, extending from the eastern African coast to the Pacific across tropical and temperate regions (Friday and Okano 2006). In China, it occurs along the coral coasts of Hainan Island (Wang 1993) and on several islets in the South China Sea (Xiong et al. 2022), where it is classified as a second-class national protected plant. Well-adapted to Hainan’s tropical monsoon climate, *C. subcordata* exhibits tolerance to high temperatures, drought, saline soils, and strong winds (Liang and Wang 2017). Genetic studies have demonstrated its remarkable adaptability to harsh environments (Chen et al. 2023). Ecologically, it functions as a windbreak and plays a crucial role in preventing coastal soil erosion (Xiong et al. 2022).

From a pharmacological perspective, the ethanol extract of *C. subcordata* has been shown to exhibit antioxidant properties by maintaining the activities of superoxide dismutase (SOD) and catalase (CAT) under drug-induced oxidative stress (Gandhimathi and Kumar 2009). This study presents the first annotation, characterization, and phylogenetic analysis of the complete chloroplast genome of *C. subcordata*.

## Materials and methods

**Figure 1.**
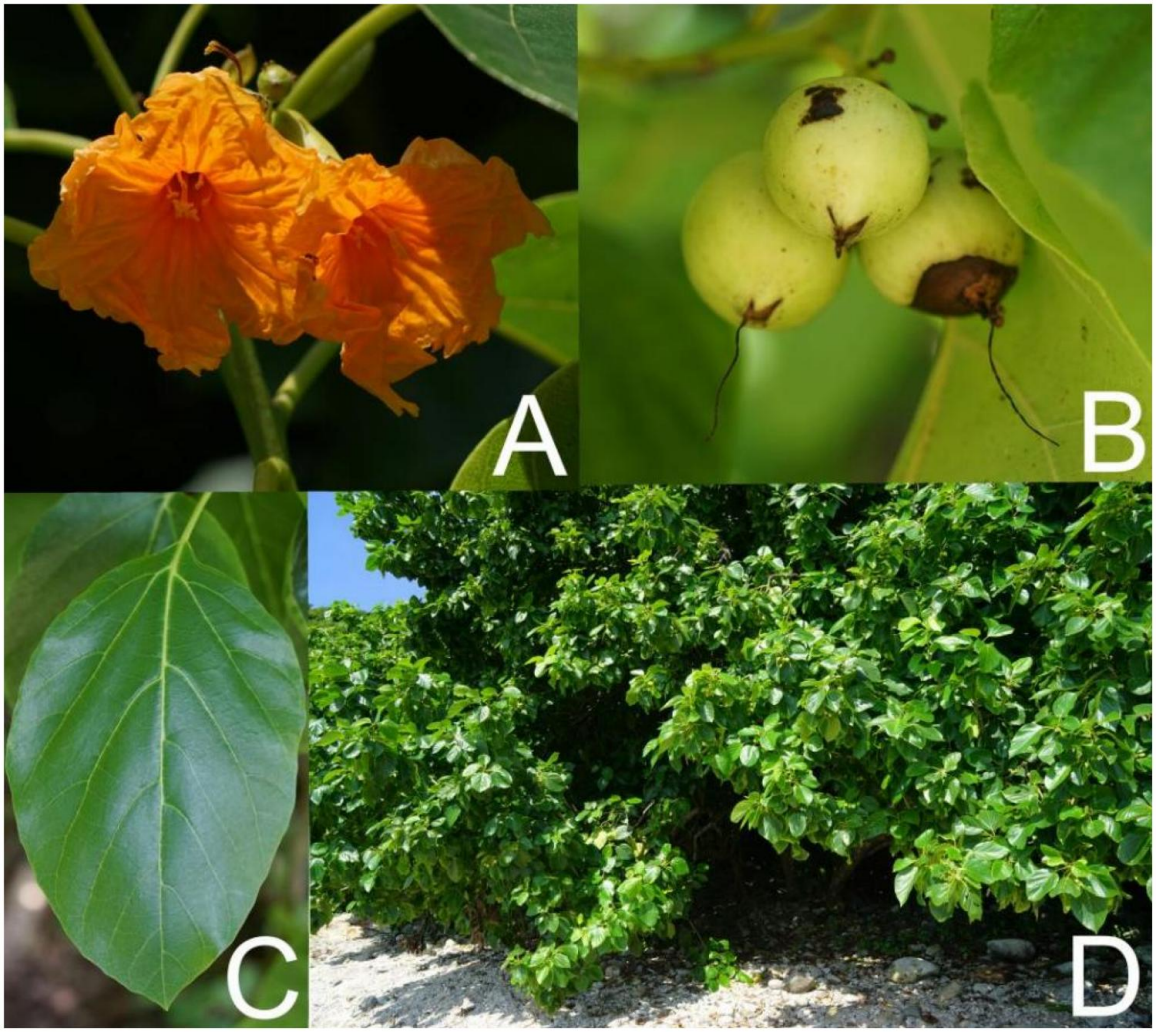
Photos of *Cordia subcordata* (photo credit: Mingzhong liu). (A) The corolla is orange, funnel-shaped, 3.4–4.5 cm in length, with a throat about 4 cm wide and orbicular, divaricate lobes. (B) The drupes are ovoid or obovoid, around 2.5 cm in size, with a corky mesocarp. (C) The leaf blade is ovate to narrowly ovate, ranging from 8–18 × 6–13 cm, with dense cottony pubescence in the vein axils on the abaxial side and some spotting on the adaxial side. (D) The tree reaches up to approximately 3 meters in height, with yellow-brown bark and glabrous branchlets.

Healthy leaves (10.11 g) of *C. subcordata* (Figure 1) were collected from Luhuitou Peninsula (109°29′53.1″E; 18°11′50.03″N), Hainan Island, China. The samples were cleaned, silica-dried, and stored at room temperature. A voucher specimen (number TOUSECO-00032) was deposited in the herbarium of Biomarker Technology Co., Qingdao, China (http://www.bmkgene.com/, contact: Jinhu Mu, mujh@biomarker.com.cn).

Genomic DNA was extracted using a modified CTAB protocol. As a modification, plant tissues preserved in silica gel were used as the DNA source. For cell lysis, a buffer containing 4% CTAB and 4 mol/L NaCl was utilized. DNA isolation was performed with chloroform-isoamyl alcohol (24:1, v/v) instead of phenol to reduce health risks. Elution was carried out using 1 × TE (Tris-EDTA) buffer rather than deionized water to enhance DNA stability. DNA quantification was conducted using a Qubit fluorometer system (Thermo Fisher Scientific, Waltham, MA, USA), which provides more accurate concentration measurements compared to the NanoDrop spectrophotometer (Schenk et al. 2023). DNA purification was performed using the Genomic DNA Purification Kit (K0512, https://store.sangon.com/). DNA quality and quantity were assessed using a NanoDrop ND-1000 Spectrophotometer (NanoDrop Technologies, Wilmington, DE, USA) and a Qubit fluorometer (Invitrogen, San Diego, CA). Sequencing libraries were prepared using the VAHTS Universal DNA Library Prep Kit (ND607-02) and sequenced on the Illumina Novaseq 6000 platform (150 bp paired-end reads) (Illumina, San Diego, CA). Quality control of raw reads was conducted using Fastp v0.20.0 (https://github.com/OpenGene/Fastp). The chloroplast genome was assembled *de novo* using SPAdes v3.14.1 (Bankevich et al. 2012) and annotated with GeSeq (Tillich et al. 2017). The genome map was visualized using OGDRAW v1.3.1 (Greiner et al. 2019). The phylogenetic analysis involved aligning the chloroplast genomes of *C. subcordata* and 24 related species (with *Dieffenbachia seguine* as an outgroup) using MAFFT v7.490 (Katoh and Standley 2013). The evolutionary relationships were analyzed using the maximum likelihood (ML) method in MEGA v6.0 (Tamura et al. 2013), with the best-fitting model (TIM+F + R5) identified through ModelFinder (Kalyaanamoorthy et al. 2017). The bootstrap value was calculated from 1,000 replicates.

## Results

**Figure 2.**
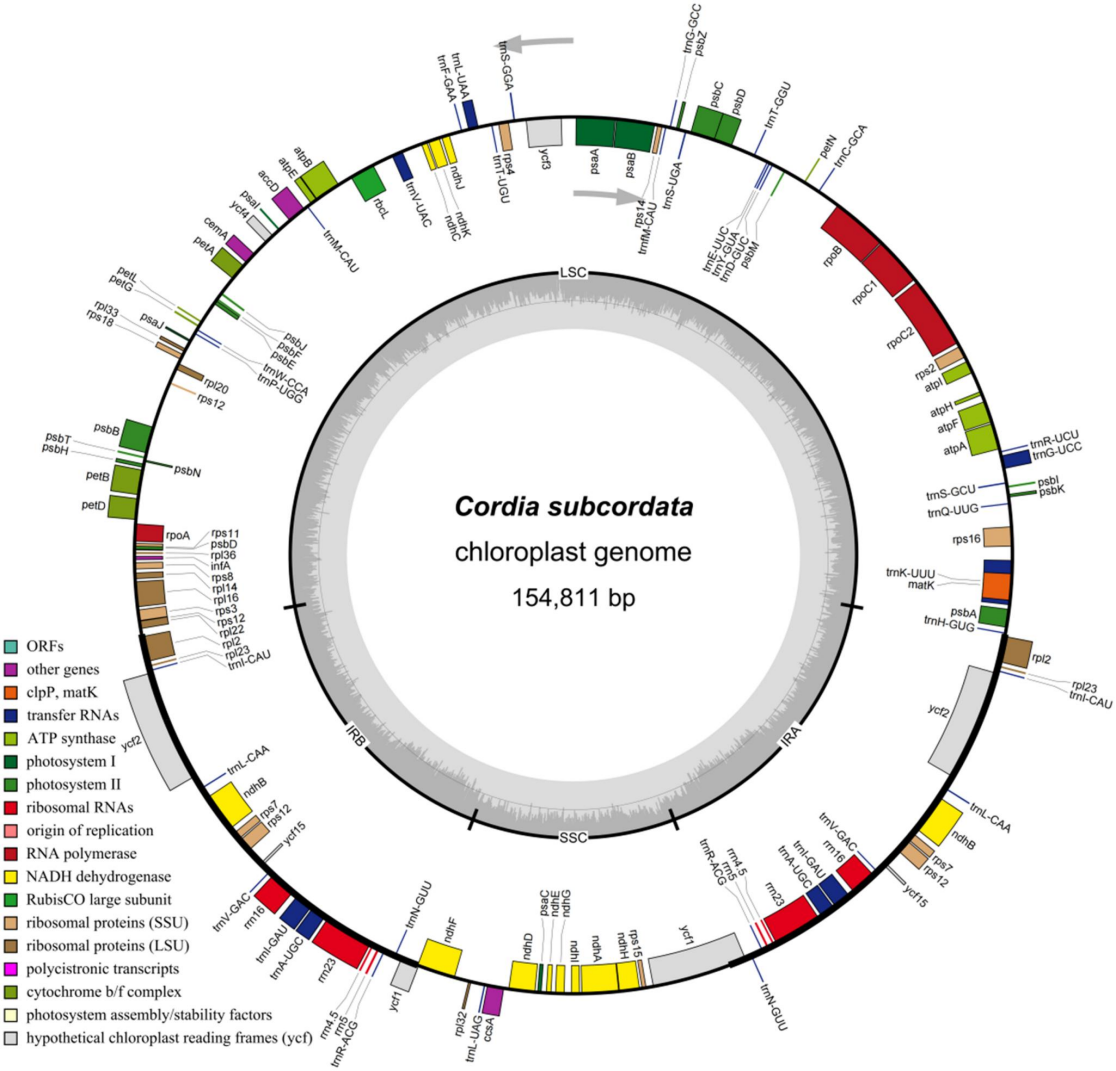
A schematic representation of the chloroplast genome of *Cordia subcordata* is shown. The genome is a typical circular structure consisting of four regions: a large single-copy (LSC) region, a small single-copy (SSC) region, and a pair of inverted repeat regions (IRA and IRB). In the innermost circle, darker gray indicates GC content, while lighter gray represents AT content. The outer circle shows the transcriptional direction of genes: those located outside the circle are transcribed counterclockwise, while those inside the circle are transcribed clockwise. Genes are color-coded according to their functional groups.

The chloroplast genome of *C. subcordata* is 154,811 bp in length, with an average sequencing depth of 7,702.901× and a minimum depth of 2,194× (Supplemental Figure S1). The circular genome (Figure 2) consists of 86,347 bp in the Large Single-Copy (LSC) region (GC content: 36.01%), 17,876 bp in the Small Single-Copy (SSC) region (GC content: 32.24%), and 25,296 bp in each of the inverted repeats (IRA and IRB) (GC content: 43.46%). The overall GC content is 38.01%. The genome contains 133 genes, including 88 protein-coding genes (PCGs), 37 transfer-RNA (tRNA) genes, and 8 ribosomal-RNA (rRNA) genes. Seven PCGs (*ndh*B, *pet*B, *pet*D, *atp*F, *rpl*16, *rps*16, *rpo*C1) and six tRNA genes (*trn*A-UGC, *trn*G-UCC, *trn*I-GAU, *trn*K-UUU, *trn*L-UAA, *trn*V-UAC) each contain one intron, while two PCGs (*clp*P, *ycf*3) contain two introns. Additionally, five PCGs (*ndh*B, *rpl*2, *ycf*1, *ycf*2, *ycf*15), seven tRNA genes, and all four rRNA genes are multi-copy genes with two copies. All tRNA genes possess a cloverleaf secondary structure. Structures of 12 cis-splicing PCGs (*rps*l6, *atp*F, *rpo*C1, *ycf*3, *pet*B, *pet*D, *rpl*l6, *rpl*2, *ndh*B, *ndh*A, *ndh*B, *rpl*2) and 1 trans-splicing gene (*rps*12) are presented in Supplemental Figures S2 and S3, respectively.

**Figure 3.**
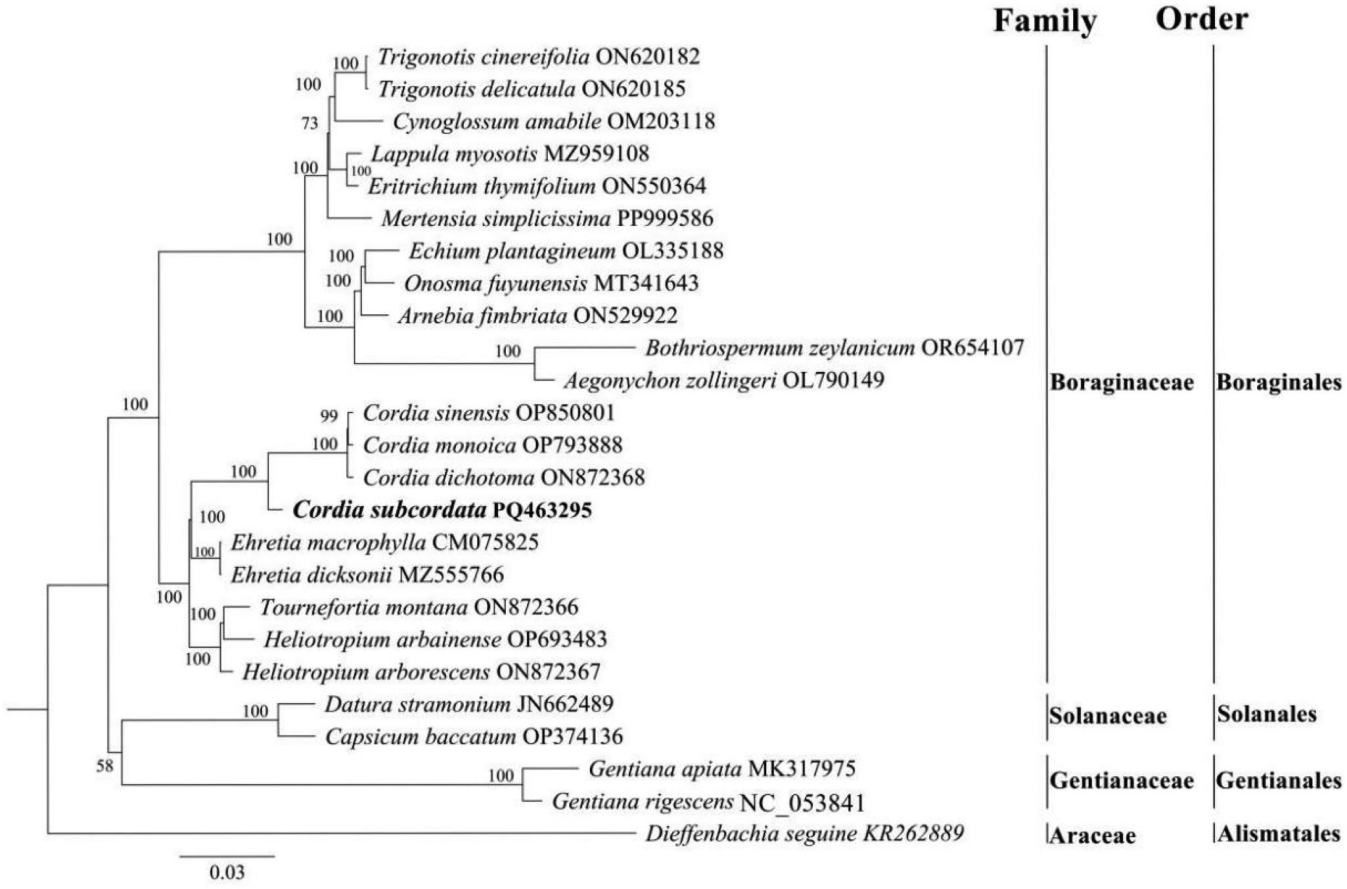
A maximum likelihood phylogenetic tree was constructed based on the complete chloroplast genome sequences of *Cordia subcordata* (PQ463295) and 24 related species, with *Dieffenbachia seguine* KR262889 (Wang et al. 2016) used as the outgroup. Numbers on nodes indicate bootstrap values with 1,000 replicates. The following sequences were used: ON620182 (Xu et al. 2023), ON620185 (Xu et al. 2023), OM203118, MZ959108 (Yan et al. 2023), ON550364, PP999586, OL335188 (Carvalho Leonardo et al. 2022a), MT341643 (He et al. 2021), ON529922 (Sun et al. 2022), OR654107, OL790149, OP850801 (Alawfi and Albokhari 2023), OP793888 (Alawfi and Albokhari 2023), ON872368 (Alawfi and Albokhari 2023), CM075825, MZ555766 (Alawfi et al. 2023), ON872366 (Li and Wei 2022), OP693483 (Alawfi et al. 2024), ON872367 (Li and Wei 2022), JN662489 (Prasad et al. 2020), OP374136, MK317975 (She et al. 2019), NC_053841 (Liang et al. 2020).

Based on the phylogenetic analysis of the complete chloroplast genomes of *C. subcordata* and 24 related species, *C. subcordata* clusters in a well-supported clade with three other species from the *Cordia* genus (*C. sinensis*, *C. monoica*, and *C. dichotoma*), with *Ehretia* species forming a sister group to the *Cordia* clade (Figure 3). The borders of the LSC, SSC, and IR regions for the 4 *Cordi*a species are compared in Figure S4, and a visualization of their chloroplast genome alignment is presented in Figure S5.

## Discussion and conclusion

*C. subcordata* is native to Malesia but has since spread widely across the Pacific and along the shores of the Indian Ocean. It thrives in coastal and lowland environments from East Africa to Polynesia (Allen 2002) and is also present in Sri Lanka, where conservation efforts have been advocated to protect its populations (Jayasuriya and Gunatilleke 2015).

The analysis of chloroplast genomes provides valuable insights into phylogenetic relationships, adaptation, plastid function evolution, and the identification of genetic markers essential for conservation and breeding (Jensen and Leister 2014). However, despite the genus *Cordia* comprising 271 species and the family Boraginaceae including 153 genera, only a limited number of species have complete chloroplast genome data available in public databases (https://www.ncbi.nlm.nih.gov/).

In this study, phylogenetic analysis of the complete chloroplast genome of *C. subcordata* alongside 24 related species enhances our understanding of evolutionary relationships within Boraginaceae. *C. subcordata* clusters with three other *Cordia* species in a strongly supported clade (bootstrap value 100%), confirming their close evolutionary ties. However, genomic data remain scarce for these related *Cordia* species. *C. sinensis*, a species distributed from Africa to the Indian subcontinent, shares ecological similarities with *C. subcordata*, thriving in seasonally dry tropical biomes (https://powo.science.kew.org/). Moreover, the genus *Cordia* is phylogenetically distinct from *Ehretia* and *Heliotropium*, underscoring the diversification within Boraginaceae. The inclusion of outgroup taxa from Solanaceae and Gentianaceae further supports the monophyly of Boraginaceae and highlights its divergence from unrelated families. The findings of this study align with previous phylogenetic research on Boraginaceae, yet they provide a more comprehensive representation of species within the family (Carvalho Leonardo et al. 2022a; Carvalho Leonardo et al. 2022b; Carvalho Leonardo et al. 2023; Wu et al. 2022; Zhao and Yu 2023). By establishing a genomic framework, this research contributes to understanding species diversification, genome evolution, and ecological adaptation in coastal environments. It also lays the foundation for further exploration of the adaptive strategies of *C. subcordata* and its relatives.

Notably, this study presents the first complete chloroplast genome of *C. subcordata*, a protected coastal species in China. The assembled genome (154,811 bp) reveals structural features such as intron-containing genes and multi-copy genes, offering novel genomic insights into Boraginaceae. Phylogenetic analysis confirms that *C. subcordata* is closely related to other *Cordia* species and distinct from *Ehretia* and *Heliotropium*, thereby clarifying taxonomic relationships and resolving evolutionary uncertainties. These findings provide critical genetic markers for conservation and serve as a valuable resource for studying coastal adaptation mechanisms, ultimately informing future efforts to protect this ecologically significant species.

## Statement

*C. subcordata* is classified as Least Concern (LC) by the International Union for Conservation of Nature (IUCN) Red List, and we have fully complied with IUCN‘s “Guidelines for Appropriate Uses of IUCN Red List Data.” Our research also adheres to the provisions of the Convention on Biological Diversity (CBD), ensuring that all activities are aligned with international biodiversity conservation principles. We have ensured compliance with the Convention on International Trade in Endangered Species of Wild Fauna and Flora (CITES) and respect all regulations regarding trade and protection of species. The species was sampled legally, with all required permits and approvals obtained. This study contributes to the broader goal of conservation by enhancing our understanding of the species, while strictly following national and international laws and ethical standards related to biodiversity conservation and research.

## Supplementary Material

Supplementary Figure3 with caption.jpg

Supplementary Figure5(1) with caption.jpg

Supplementary Figure2 with caption.jpg

Supplementary Figure4 with caption.jpg

Supplementary Figure5(2) with caption.jpg

Supplementary Figure5(3) with caption.jpg

Supplementary Figure1 with caption.jpg

## Data Availability

The data that support the finding of this study are openly available in GenBank of NCBl at https://www.ncbi.nlm.nih.gov, reference number PQ463295.1 for *C. subcordata*. The associated BioProject, BioSample, and SRA numbers are PRJNA1191212, SAMN45073647, and SRR31593877 respectively.

## References

[CIT0001] Alawfi MS, Albokhari EJ. 2023. Comparative chloroplast genomics reveals a unique gene inversion in two *Cordia* trees (Cordiaceae). Forests. 14(9):1778. doi:10.3390/f14091778.

[CIT0002] Alawfi MS, Alzahrani DA, Albokhari EJ. 2023. Complete chloroplast genome sequences of two *Ehretia* trees (*Ehretia cymosa* and *Ehretia obtusifolia*): Genome structures and phylogenetic analysis. Forests. 14(7):1486. doi:10.3390/f14071486.

[CIT0003] Alawfi MS, Alzahrani DA, Albokhari EJ. 2024. Complete plastome genomes of three medicinal heliotropiaceae species: comparative analyses and phylogenetic relationships. BMC Plant Biol. 24(1):654. doi:10.1186/s12870-024-05388-8. 38987665 PMC11234707

[CIT0004] Allen JA. 2002. *Cordia subcordata* Lam. Tropical tree seed manual: Part II, species descriptions. Washington, DC: U.S. Department of Agriculture. vol.712; p. 418–419. doi:10.1093/aob/mch046

[CIT0005] Bankevich A, Nurk S, Antipov D, Gurevich AA, Dvorkin M, Kulikov AS, Lesin VM, Nikolenko SI, Pham S, Prjibelski AD, et al. 2012. SPAdes: a new genome assembly algorithm and its applications to single-cell sequencing. J Comput Biol. 19(5):455–477. doi:10.1089/cmb.2012.0021. 22506599 PMC3342519

[CIT0006] Bawa KS. 1974. Breeding systems of tree species of a lowland tropical community. Evolution. 28(1):85–92. doi:10.2307/2407241. 28563035

[CIT0007] Carvalho Leonardo I, Alberti A, Denoeud F, Barreto Crespo MT, Capelo J, Bustos Gaspar F. 2023. The complete plastome of *Glandora prostrata* subsp. lusitanica (Samp.) DC Thomas (Boraginaceae), the first chloroplast genome belonging to the *Glandora* genus. Mitochondrial DNA B Resour. 8(2):270–273. doi:10.1080/23802359.2023.2175976. 36816053 PMC9937008

[CIT0008] Carvalho Leonardo I, Barreto Crespo MT, Capelo J, Bustos Gaspar F. 2022a. The complete plastome of *Echium plantagineum* L.(Boraginaceae), the first chloroplast genome belonging to the *Echium* genus. Mitochondrial DNA B Resour. 7(6):1154–1156. doi:10.1080/23802359.2022.2087559. 35783061 PMC9245987

[CIT0009] Carvalho Leonardo I, Barreto Crespo MT, Capelo J, Bustos Gaspar F. 2022b. The complete plastome of *Nonea vesicaria* (L.) Rchb. (Boraginaceae), the first chloroplast genome belonging to the *Nonea* genus. Mitochondrial DNA B Resour. 7(7):1302–1304. doi:10.1080/23802359.2022.2095233. 35874278 PMC9297717

[CIT0010] Chen YL, Wang ZF, Jian SG, Liao HM, Liu DM. 2023. Genome assembly of *Cordia subcordata*, a coastal protection species in tropical coral islands. Int J Mol Sci. 24(22):16273. doi:10.3390/ijms242216273. 38003462 PMC10671804

[CIT0011] Friday JB, Okano D. 2006. *Cordia subcordata* (kou). Species Profiles for Pacific Island Agroforestry. 3:1.

[CIT0012] Gandhimathi R, Kumar AS. 2009. Evaluation of antioxidant activity of *Cordia Subcordata* Lam. against carbon tetrachloride (CCl4) induced erythrocyte damage in rats. Pharmacol. 2:720–727.

[CIT0013] Greiner S, Lehwark P, Bock R. 2019. OrganellarGenomeDRAW (OGDRAW) version 1.3. 1: expanded toolkit for the graphical visualization of organellar genomes. Nucleic Acids Res. 47(W1):W59–W64. doi:10.1093/nar/gkz238. 30949694 PMC6602502

[CIT0014] He Y, Xu X, Liu Q. 2021. The complete chloroplast genome of *Onosma fuyunensis* Y. He & Q.R. Liu and its phylogenetic analysis. Mitochondrial DNA B Resour. 6(11):3142–3143. doi:10.1080/23802359.2020.1861567. 34746385 PMC8567896

[CIT0015] Jayasuriya AHM, Gunatilleke IAUN. 2015. A refugium for *Cordia subcordata* (Boraginaceae), a very rare and endangered plant in Sri Lanka and strategies for its conservation. Ceylon J. Sci. (Biol. Sci.). 44(1):67–70. doi:10.4038/cjsbs.v44i1.7343.

[CIT0016] Jensen PE, Leister D. 2014. Chloroplast evolution, structure and functions. F1000Prime Rep. 6:40. doi:10.12703/P6-40. 24991417 PMC4075315

[CIT0017] Kalyaanamoorthy S, Minh BQ, Wong TKF, von Haeseler A, Jermiin LS. 2017. ModelFinder: fast model selection for accurate phylogenetic estimates. Nat Methods. 14(6):587–589. doi:10.1038/nmeth.4285. 28481363 PMC5453245

[CIT0018] Katoh K, Standley DM. 2013. MAFFT multiple sequence alignment software version 7: improvements in performance and usability. Mol Biol Evol. 30(4):772–780. doi:10.1093/molbev/mst010. 23329690 PMC3603318

[CIT0019] Li Q, Wei R. 2022. Comparison of Boraginales plastomes: insights into codon usage bias, adaptive evolution, and phylogenetic relationships. Diversity. 14(12):1104. doi:10.3390/d14121104.

[CIT0020] Liang Y, Meng X, Mu Z, Qian J, Duan B, Xu L. 2020. The complete chloroplast genome and phylogenetic analysis of *Gentiana manshurica* Kitag from China. Mitochondrial DNA Part B. 5(2):1625–1626. doi:10.1080/23802359.2020.1745107.

[CIT0021] Liang Z, Wang D. 2017. Sea breeze and precipitation over Hainan Island. Quart J Royal Meteoro Soc. 143(702):137–151. doi:10.1002/qj.2952.

[CIT0022] Matias EFF, Alves EF, do Nascimento Silva MK, de Alencar Carvalho VR, Coutinho HDM, da Costa JGM. 2015. The genus *Cordia*: botanists, ethno, chemical and pharmacological aspects. Revista Brasileira de Farmacognosia. 25(5):542–552. doi:10.1016/j.bjp.2015.05.012.

[CIT0023] Opler PA, Baker HG, Frankie GW. 1975. Reproductive biology of some Costa Rican *Cordia* species (Boraginaceae). Biotropica. 7(4):234–247. doi:10.2307/2989736.

[CIT0024] Prasad G, Pratap G, Marimuthu S, Prasad S, Rao G, Mangal A, Srikanth N. 2020. Molecular identification and next-generation sequence analysis of interspecies genetic variations among three varieties of datura. Phcog Res. 12(2):158. doi:10.4103/pr.pr_101_19.

[CIT0025] Schenk JJ, Becklund LE, Carey SJ, Fabre PP. 2023. What is the “modified” CTAB protocol? Characterizing modifications to the CTAB DNA extraction protocol. Appl Plant Sci. 11(3):e11517. doi:10.1002/aps3.11517. 37342162 PMC10278931

[CIT0026] She RX, Li W, Xie X, Liu M, Wang L, Khan H, Ullah I, Zhao P. 2019. The complete chloroplast genome of *Gentiana apiata* (Gentianaceae), an endemic species to Qinba Mountain in China. Mitochondrial DNA Part B. 4(1):1609–1610. doi:10.1080/23802359.2019.1604099. PMC7706864 33365641

[CIT0027] Sun J, Wang S, Wang Y, Wang R, Liu K, Li E, Qiao P, Shi L, Dong W, Huang L, et al. 2022. Phylogenomics and genetic diversity of *Arnebiae Radix* and its allies (*Arnebia*, Boraginaceae) in China. Front Plant Sci. 13:920826. doi:10.3389/fpls.2022.920826. 35755641 PMC9218939

[CIT0028] Tamura K, Stecher G, Peterson D, Filipski A, Kumar S. 2013. MEGA6: molecular evolutionary genetics analysis version 6.0. Mol Biol Evol. 30(12):2725–2729. doi:10.1093/molbev/mst197. 24132122 PMC3840312

[CIT0029] Tillich M, Lehwark P, Pellizzer T, Ulbricht-Jones ES, Fischer A, Bock R, Greiner S. 2017. GeSeq-versatile and accurate annotation of organelle genomes. Nucleic Acids Res. 45(W1):W6–W11. J doi:10.1093/nar/gkx391. 28486635 PMC5570176

[CIT0030] Verdcourt B. 1991. Flora of Tropical East Africa-Boraginaceae (1st ed.). Routledge. doi:10.1201/9780203755846.

[CIT0031] Wang B, Han L, Chen C, Wang Z. 2016. The complete chloroplast genome sequence of *Dieffenbachia seguine* (Araceae). Mitochondrial DNA A DNA Mapp Seq Anal. 27(4):2913–2914. doi:10.3109/19401736.2015.1060436. 26153749

[CIT0032] Wang W. 1993. An enumeration of the Boraginaceous plants collected by H. smith from China during 1921-22, 1924 and 1934. Bull. Bot. Res. 13:1–10.

[CIT0033] Wang X, Wen M, Wu M, Zhang D. 2020. *Cordia subcordata* (Boraginaceae), a distylous species on oceanic coral islands, is self-compatible and pollinated by a passerine bird. Pl Ecol Evol. 153(3):361–372. doi:10.5091/plecevo.2020.1757.

[CIT0034] Wu JH, Li HM, Lei JM, Liang ZR. 2022. The complete chloroplast genome sequence of *Trigonotis peduncularis* (Boraginaceae). Mitochondrial DNA B Resour. 7(3):456–457. doi:10.1080/23802359.2022.2048212. 35274042 PMC8903795

[CIT0035] Xiong Y, Chen X, Wu K, et al. 2022. Shoot organogenesis and plant regeneration in *Cordia subcordata* Lam. In Vitro Cellular & Developmental Biology-Plant. 58:392–398. doi:10.1007/s11627-021-10233-w

[CIT0036] Xu XM, Liu DH, Zhu SX, Wang ZL, Wei Z, Liu QR. 2023. Phylogeny of Trigonotis in China—with a special reference to its nutlet morphology and plastid genome. Plant Divers. 45(4):409–421. doi:10.1016/j.pld.2023.03.004. 37601540 PMC10435912

[CIT0037] Yan S, Wang T, Wang Z, Ren W, Liu C, Ma W, Dong S. 2023. The chloroplast genome of *Lappula myosotis* V. Wolf, a medicinal species. Mitochondrial DNA B Resour. 8(1):30–33. doi:10.1080/23802359.2022.2158692. 36620320 PMC9815226

[CIT0038] Zhao ZN, Yu X. 2023. The complete chloroplast genome of *Cynoglossum amabile* Stapf & JR Drumm., 1906 (Boraginaceae), a traditional Chinese herbal medicine. Mitochondrial DNA B Resour. 8(1):52–56. doi:10.1080/23802359.2022.2160219. 36620318 PMC9815245

