# Supplementary figures and images for: Characterization and phylogenetic analysis of the complete chloroplast genome of Cordia subcordata Lamarck 1899, a protected plant in China
Source: Mitochondrial DNA B Resour. 2025 Jun 20;10(7):631–6. doi: 10.1080/23802359.2025.2519220 (PMC12207770; doi:10.1080/23802359.2025.2519220)

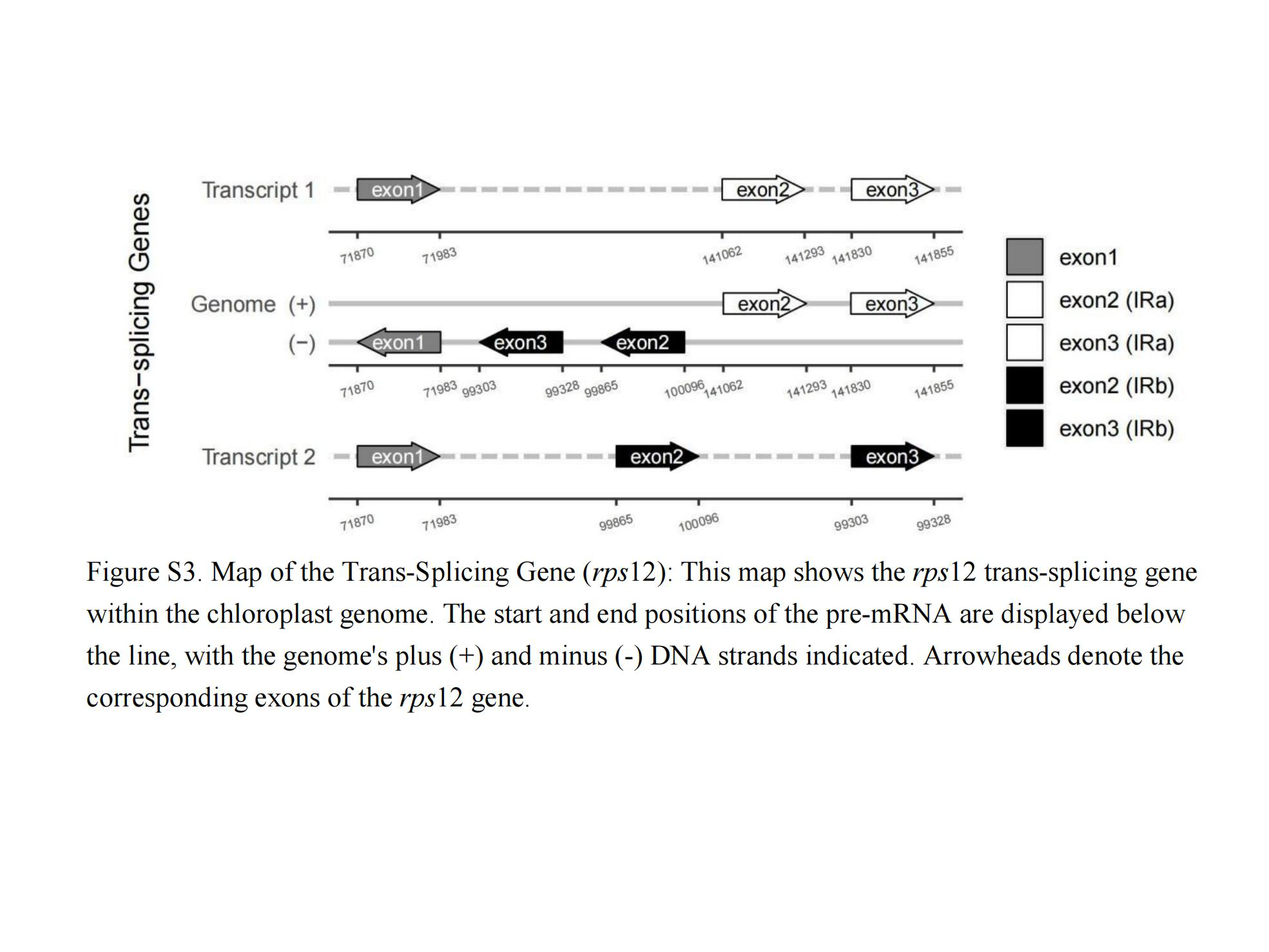

Supplement: Supplementary Figure3 with caption.jpg [file TMDN_A_2519220_SM5743.jpg]

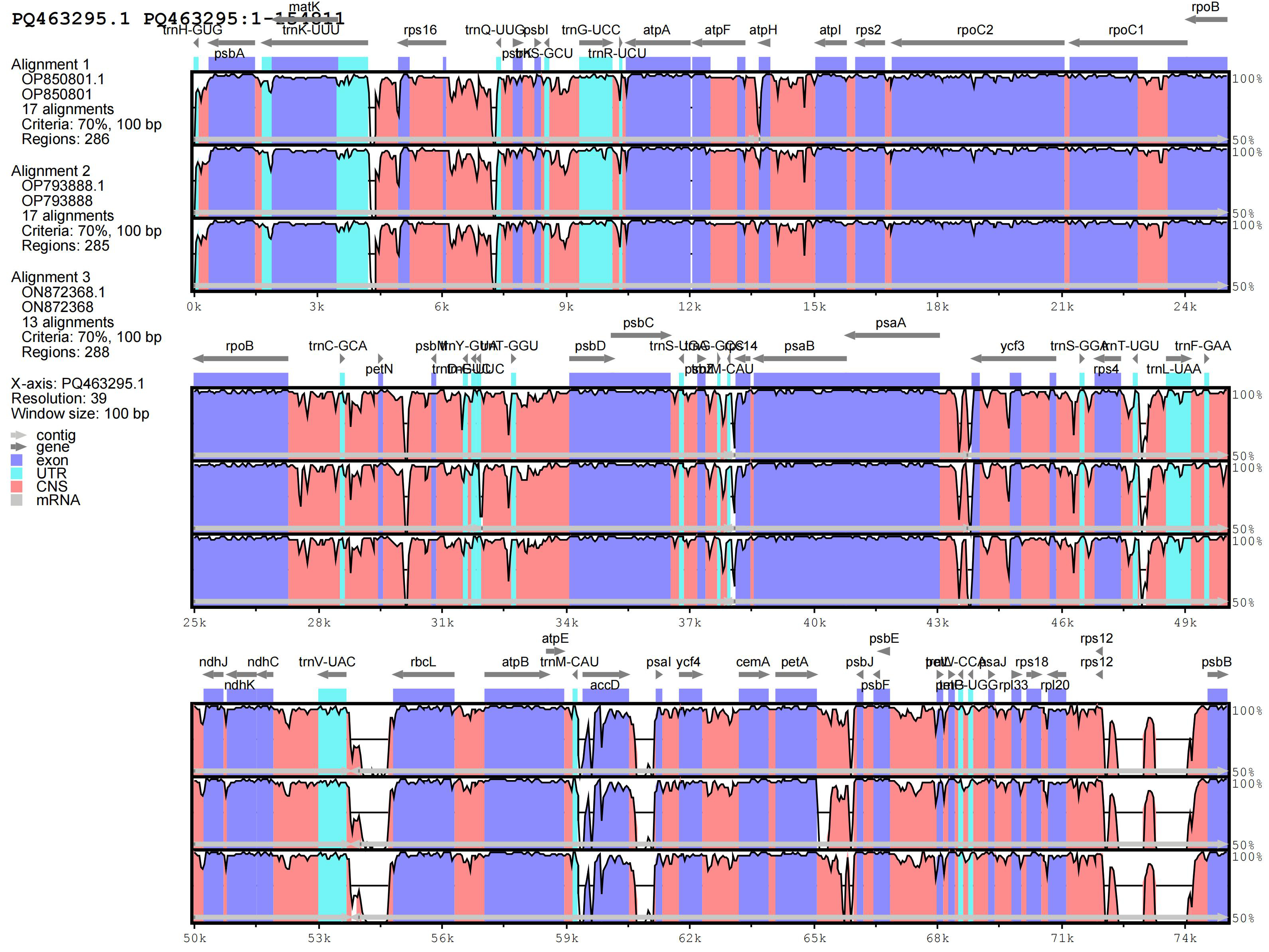

Supplement: Supplementary Figure5(1) with caption.jpg [file TMDN_A_2519220_SM5742.jpg]

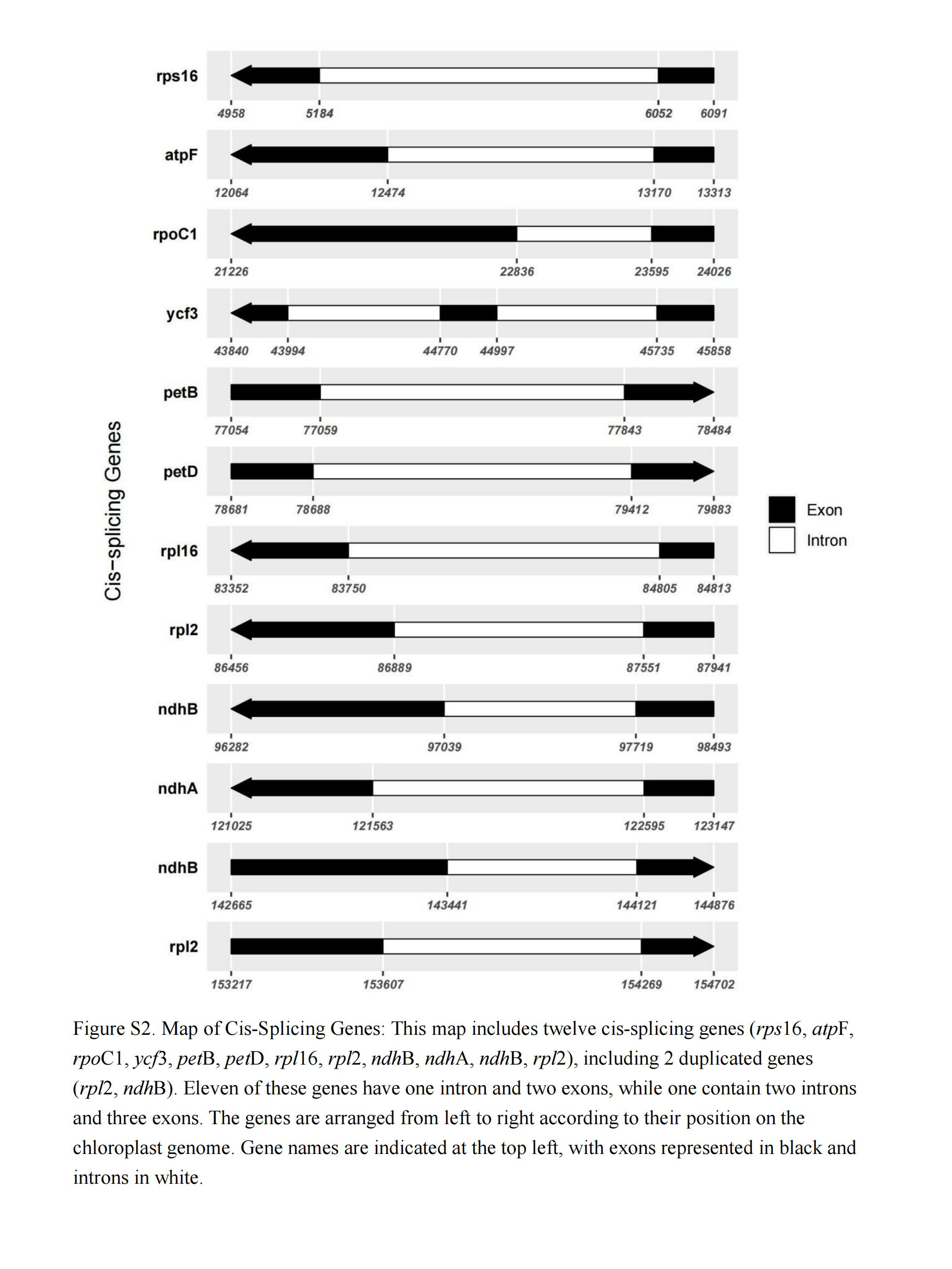

Supplement: Supplementary Figure2 with caption.jpg [file TMDN_A_2519220_SM5741.jpg]

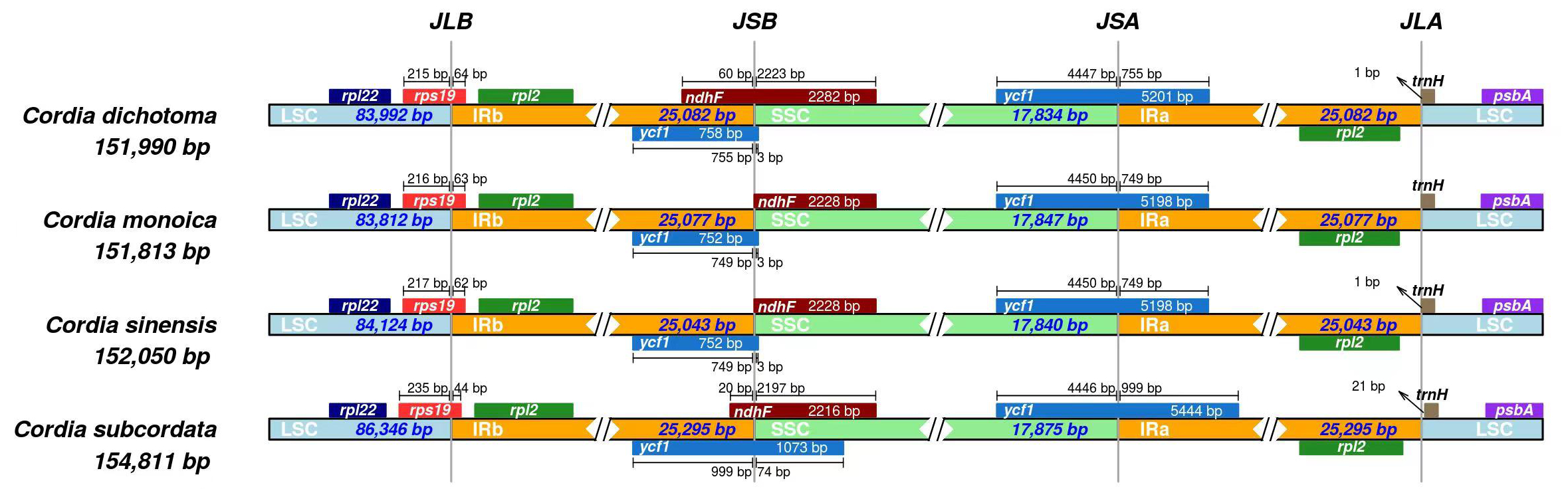

Supplement: Supplementary Figure4 with caption.jpg [file TMDN_A_2519220_SM5740.jpg]

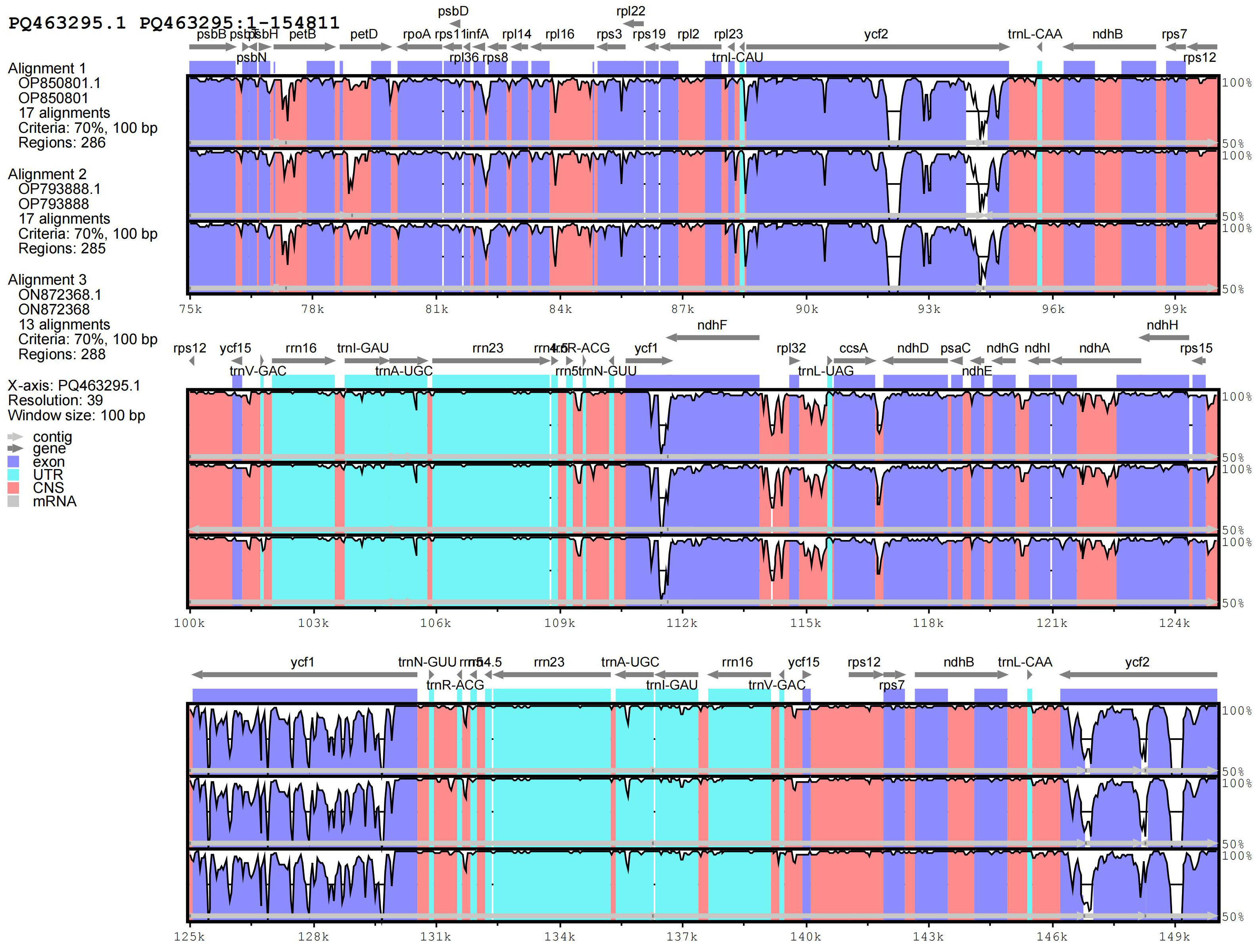

Supplement: Supplementary Figure5(2) with caption.jpg [file TMDN_A_2519220_SM5739.jpg]

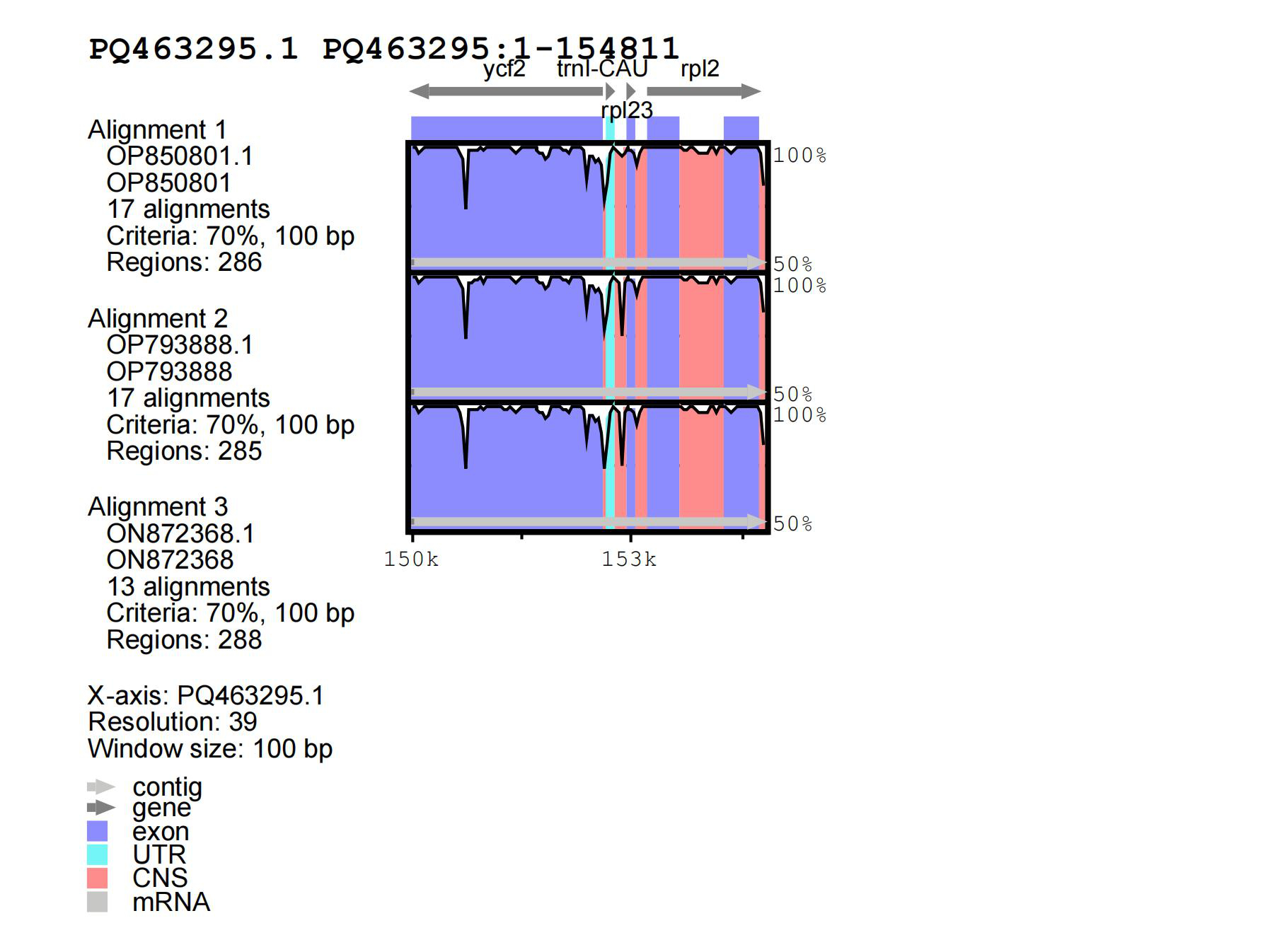

Supplement: Supplementary Figure5(3) with caption.jpg [file TMDN_A_2519220_SM5738.jpg]

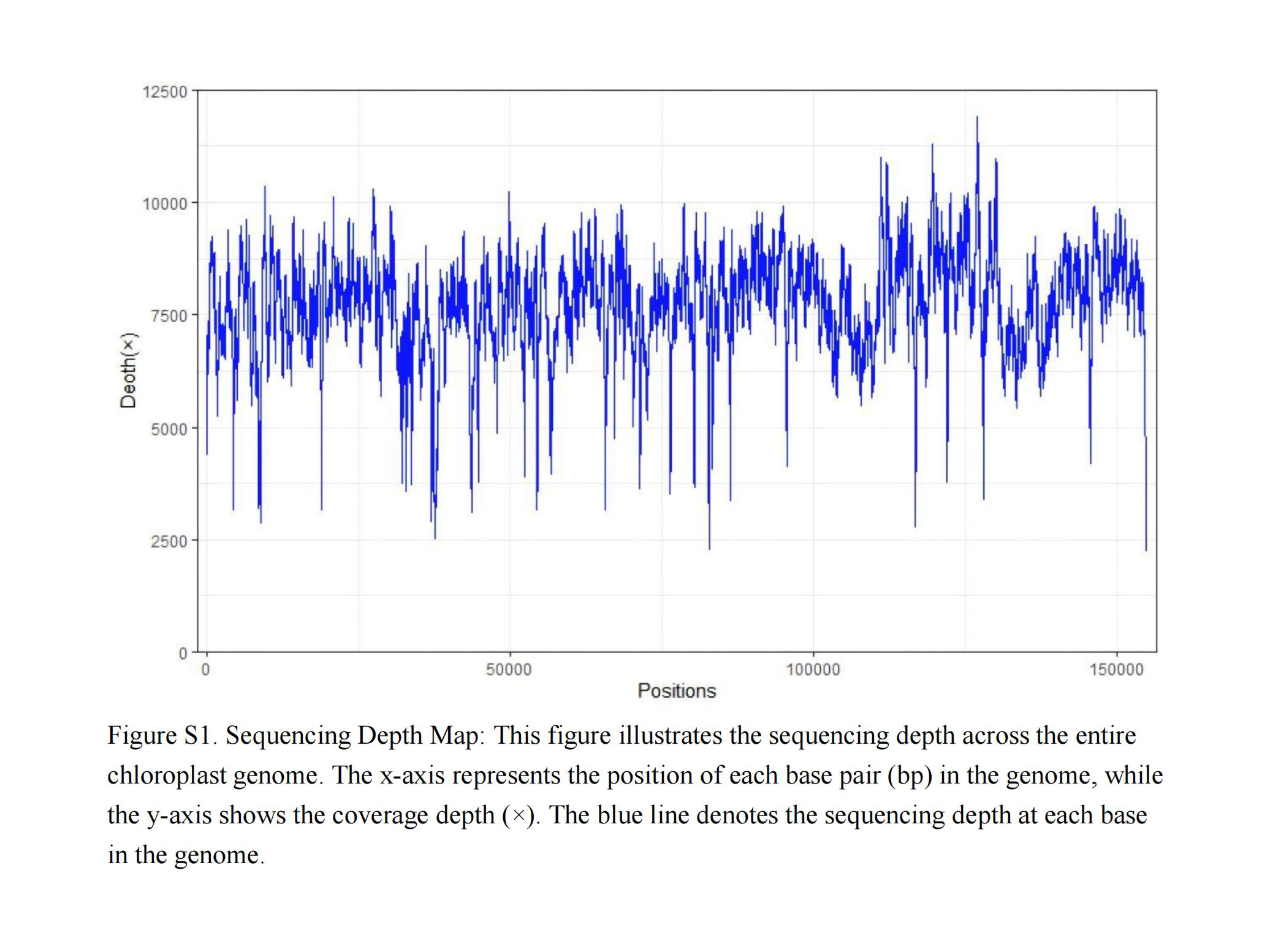

Supplement: Supplementary Figure1 with caption.jpg [file TMDN_A_2519220_SM5737.jpg]
